# Childhood Adversity and Affective Touch Perception: A Comparison of United Kingdom Care Leavers and Non-care Leavers

**DOI:** 10.3389/fpsyg.2020.557171

**Published:** 2020-11-10

**Authors:** Shaunna L. Devine, Susannah C. Walker, Adarsh Makdani, Elizabeth R. Stockton, Martyn J. McFarquhar, Francis P. McGlone, Paula D. Trotter

**Affiliations:** ^1^Research Centre for Brain and Behaviour, Liverpool John Moores University, Liverpool, United Kingdom; ^2^Division of Neuroscience and Experimental Psychology, The University of Manchester, Manchester, United Kingdom; ^3^Institute of Psychology, Health and Society, University of Liverpool, Liverpool, United Kingdom

**Keywords:** touch, childhood adversity, perception, neglect, C-tactile afferent, affective touch

## Abstract

In the United Kingdom, the most common reasons for a child to come under the care of social services are neglect and abuse. Such early childhood adversity is a risk factor for social-isolation and poor mental health in adulthood. Touch is a key channel for nurturing interactions, and previous studies have shown links between early somatosensory input, experience dependent neural plasticity, and later life emotional functioning. The aim of the present study was to test the relationship between childhood neglect/abuse and later life experiences, attitudes, and hedonic ratings of affective touch. Here, affective touch is defined as low force, dynamic touch which C-Tactile afferents (CTs) respond optimally to. We hypothesized that a childhood lacking in early nurturing tactile stimulation would be associated with reduced sensitivity to socially relevant affective touch in adulthood. To test this, 19 care leavers (average 9.32 ± 3.70 years in foster care) and 32 non-care leavers were recruited through opportunity sampling (mean age = 21.25 ± 1.74 years). Participants completed a range of psychophysical somatosensory tests. First, they rated the pleasantness of CT-optimal (3 cm/s) and non-CT-optimal (0.3 and 30 cm/s) stroking touch applied to their forearm, both robotically and by an experimenter. They also made vicarious ratings of the anticipated pleasantness of social tactile interactions depicted in a series of videos. Finally, they filled in the Childhood Trauma Questionnaire (CTQ) and the Touch Experiences and Attitudes Questionnaire (TEAQ). As expected, care leavers reported significantly higher levels of childhood trauma than the control group. They also reported significantly lower levels of positive childhood touch compared to non-care leavers, but their attitudes and experiences of current intimate and affiliative touch did not differ. Across all psychophysical tests, care leavers showed specific reduction in sensitivity to the affective value of CT targeted 3 cm/s touch. The results of this study support the hypothesis that a lack of nurturing touch in early developmental periods leads to blunted sensitivity to the specific social value of affective touch. Future research should investigate the neural and physiological mechanisms underlying the observed effect.

## Introduction

In the United Kingdom, the most common reasons for a child to come under the care of social services are neglect and abuse (Department for Education, 2019). That is, a failure to provide appropriate physical and emotional care and/or exposure to deliberate physical and emotional harm (NSPCC, 2019). In the long term, such childhood adversity is associated with poor mental health and risky behaviors (McCann et al., 1996; Ben-Ami and Baker, 2012; McLaughlin and Sheridan, 2016; Duffy et al., 2018; Briere and Elliott, 2019). Indeed, increased frequency of adverse events in childhood leads to a higher probability of later life psychopathology (Evans et al., 2013; McLaughlin and Sheridan, 2016).

Touch is a key sensory channel for parental-infant interactions (Hofer, 1994; Walker and McGlone, 2013; McGlone et al., 2017; Van Puyvelde et al., 2019b; Montirosso and McGlone, 2020). Indeed, a range of studies have shown clear links between early nurturing tactile interactions, experience dependent neural plasticity, hypothalamic-pituitary-adrenal (HPA) axis development, and later life social and emotional functioning (Levine, 2001; Meaney, 2001; Champagne et al., 2008; Franklin et al., 2011; Walker and McGlone, 2013; Walker et al., 2017a; Van Puyvelde et al., 2019a,b), with a paucity of early nurturing touch having adverse consequences (see Walker and McGlone, 2013; Bales et al., 2018 for reviews). In rodents, parental sensory stimulation largely consists of licking and grooming, huddling, and playing, all of which involve significant somatosensory stimulation. Levels of parental care vary between individuals, with those receiving high levels of contact showing a greater density of connections within somatosensory cortex (Seelke et al., 2016).

A distinction between the discriminative and affective functions of cutaneous senses has long been recognized for pain, where discrete classes of afferent nerves elicit different perceptual and emotional states (Bishop and Landau, 1958; Cross, 1994; Ploner et al., 2002; McGlone and Reilly, 2010). However, it is only with the more recent identification and characterization of a class of unmyelinated, low-threshold mechanoreceptors in the hairy skin of humans that the existence of a dedicated pathway for affective touch has been considered (Nordin, 1990; Vallbo et al., 1993, 1999; Löken et al., 2009). While the discriminative properties of touch are signaled by thickly myelinated fast conducting Aβ afferents projecting to primary somatosensory cortex, the affective components of touch are conveyed by a subclass of C-type fibers, named C-Tactile afferents (CTs), which project to the insular cortex (Olausson et al., 2002; McGlone et al., 2014). CT afferents are force, velocity, and temperature tuned, responding preferentially to a gentle, stroking, skin temperature touch of between 1 and 10 cm/s.

Their peripheral response characteristics, coupled with central projections to affective rather than primary sensory regions, has led to the hypothesis that the CT system evolved to signal socially relevant and rewarding touch (Löken et al., 2009; Morrison et al., 2010; Olausson et al., 2010; Ackerley et al., 2014). Indirect support for this putative social function comes from studies where, when asked to caress their infant, parents spontaneously delivered touch within the CT optimal range (Croy et al., 2016a; Van Puyvelde et al., 2019a,b; Bytomski et al., 2020).

Consistent with the CT system being active in early infancy and thus able to influence early brain development, a recent study reported that in 2-month-old infants, as in adults, greater activation was elicited in the insular cortex in response to CT optimal than to faster, non-CT optimal touch (Jönsson et al., 2018). Indeed, in term-infants just a few days old, gentle stroking has been found to lead to activation in the posterior insular cortex (Tuulari et al., 2017). In a psychophysical test, children as young as 5 years displayed a preference for CT optimal velocities of stroking touch (Croy et al., 2019).

Children raised in institutions experience reduced one-to-one caregiving. This deficit in nurturing stimulation is associated with an increased risk of neural and behavioral dysfunction (Tottenham, 2012). However, other forms of early social deprivation, such as childhood maltreatment or low socioeconomic status, are also strongly associated with structural and functional changes in association cortex and limbic regions underpinning behavioral control and emotion regulation (Hart and Rubia, 2012; McLaughlin et al., 2014).

While neural changes in sensory processing have not been widely reported in these groups, there is clear behavioral evidence of abnormalities in tactile processing during childhood (Cermak and Daunhauer, 1997; Cermak and Groza, 1998; Wilbarger et al., 2010). This can manifest as hyper-reactivity, leading the child to seek intense tactile experiences or hypo-reactivity, resulting in withdrawal from and avoidance of touch and textures that are typically perceived as pleasant or benign (Cermak and Groza, 1998). These abnormal sensory reactions can have a detrimental effect on the child’s experience of typical nurturing interactions with their mother or caregivers (Cermak and Daunhauer, 1997); rejections can manifest latterly as uncooperative and impulsive behavior (Cermak and Groza, 1998). The longer a child is raised in a suboptimal nurturing environment, the greater the impact on development and behavior (Lin et al., 2005; Wilbarger et al., 2010). For example, children spending 12 months or longer in institutionalized care showed significantly more aberrant responses to light touch (both tactile seeking and aversion) than those raised predominantly in foster care or with their biological parents (Wilbarger et al., 2010).

In England, the majority of children in care are fostered (Department for Education. England, 2017) and evidence indicates that, on average, they have a more positive developmental trajectory than those raised in institutions (Wilbarger et al., 2010). Indeed, United Kingdom fostering regulations state “carers should provide a level of care, including physical affection, which is designed to demonstrate warmth, friendliness, and positive regard for children” (Fostering Services, 2011). However, guidance from local authorities and independent fostering agencies frequently warns carers that showing physical affection toward children could be misinterpreted and put them at risk (Narey and Owers, 2018). Thus, having been taken into care due to neglect and/or abuse this group, even when fostered, are likely to experience reduced nurturing contact in comparison to peers who have never been in care. Care leavers frequently experience social-exclusion in adulthood (Stein, 2006; Atkinson and Hyde, 2019), thus it seems plausible that a lack of nurturing touch during early critical or sensitive periods has significant consequences for later life social functioning. Initial evidence for this is provided by the significant negative relationship between childhood adversity and later life touch attitudes and experiences reported by Trotter et al. (2018b). However, this study is limited in that only 13% of participants reported high levels of childhood adversity, and the sole measure of tactile processing was self-report.

The aim of the present study was to investigate whether adults who have experienced childhood neglect and/or abuse show abnormal responses to socially relevant affective touch in adulthood. Thus, we recruited young adults who have grown up in United Kingdom foster care and compared their ratings of directly and vicariously experienced touch to a group of age matched peers, raised in a typical family environment. We used psychophysical techniques to deliver dynamic tactile stimuli. Some velocities were targeted to optimally activate CTs (1–10 cm/s) whereas other, faster and slower strokes fell outside the CT optimal range (Löken et al., 2009). On hairy skin sites, CT targeted touch is reliably rated as more pleasant than faster and slower velocities (Essick et al., 1999, 2010). To consider top down effects on these ratings, touch was delivered both in a social condition, by the experimenter, and a non-social condition, using an automated robot (Triscoli et al., 2013). For the vicarious touch ratings, participants viewed a series of short videos depicting one adult touching another at various upper body sites. In the general population, we have previously shown, ratings of these stimuli have the same relationship between velocity and anticipated pleasantness as directly felt touch (Walker et al., 2017b). We hypothesized that the care leavers would report higher levels of childhood trauma, have more negative attitudes to touch and show blunted psychophysical ratings to CT targeted touch.

## Materials and Methods

### Participants

Fifty-one participants (eight males) aged 18–30 (Mean = 21.25, *SD* ± 1.74) took part in the study. Nineteen were care leavers (five males, mean age = 21.47, *SD* ± 2.04), defined as having spent a minimum of 1 year in United Kingdom foster care, recruited *via* gatekeepers, social services, and social media. Mean age at entering care was 10.53 years (*SD* ± 3.26). Mean duration in care was 9.32 years (*SD* ± 3.70). The comparison group comprised 32 non-care leavers (three males, mean age 21.13, *SD* ± 1.56), recruited *via* Liverpool John Moores University. Participants were excluded from participating if they had any skin condition affecting their arms or any neurological damage to this area. Prior to recruitment, the study was approved by Liverpool John Moores University Psychology Research Ethics committee. All participants gave written informed consent.

### Measures

#### Childhood Trauma Questionnaire

The Childhood Trauma Questionnaire (CTQ) is a 28-item retrospective self-report questionnaire designed to assess negative childhood experiences using five subscales: (1) emotional neglect (e.g., “I felt loved”), (2) emotional abuse (e.g., “People in my family said hurtful or insulting things to me”), (3) physical neglect (e.g., “I didn’t have enough to eat”), (4) physical abuse (e.g., “I was punished with a belt, a board, a cord, or some other hard object”), and (5) sexual abuse (e.g., “Someone tried to make me do sexual things or watch sexual things”; Bernstein et al., 1998). These five types of experiences are each assessed by five items; three additional items assess tendencies of respondents to minimize or deny abuse experiences. Respondents rate the truth of each statement on a five-point Likert Scale (1 = “never true” to 5 = “very often true”). Thus, scores on each subscale range from 5 to 25, some items are reverse scored and high scores indicate more negative experiences. The CTQ has high internal consistency (Cronbach’s *α* = 0.95; Strand et al., 2005).

#### Touch Experiences and Attitudes Questionnaire

The Touch Experiences and Attitudes Questionnaire (TEAQ) is a 57-item self-report questionnaire designed to measure experiences and attitudes toward touch across the lifespan using six subscales: friends and family touch (FFT), current intimate touch (CIT), childhood touch, attitude to self-care (ASC), attitude to intimate touch (AIT), and attitude to unfamiliar touch (AUT; Trotter et al., 2018b). Statements such as “I often have my skin stroked” are rated on a five-point Likert Scale (1 = “Disagree strongly” to 5 = “Agree strongly”) with high scores indicating more positive attitudes toward and experiences of touch. The TEAQ has high internal consistency (Cronbach’s *α* = 0.78–0.92; Trotter et al., 2018b).

#### Rotary Tactile Stimulator

An rotary tactile stimulator (RTS; Dancer Design, St Helens, United Kingdom), with a soft brush (head width 5 cm) attached, was used to deliver controlled brush strokes to the ventral surface of the left forearm. Participants experienced three velocities of touch (0.3, 3, and 30 cm/s), three times each in a pseudorandom order. On each of nine trials, the RTS delivered a single stroke in a proximal to distal direction, over an aperture of approximately 8 cm, at a force of 0.3 N. Participants were asked to close their eyes and turn their head away while the touch was administered. After each trial, participants were presented with a fresh 20 cm long, printed, visual analogue scale with anchors −10 = unpleasant, 0 = neutral, and + 10 = pleasant. The participant drew a mark on the paper scale to rate the perceived pleasantness of the touch they had just experienced. After they had made their rating, the participant indicated to the experimenter they were ready to progress, and the next trial was initiated.

#### Human Touch

Participants received manual strokes to the ventral surface of their left forearm, delivered by the experimenter using their dominant hand on which, for consistency of temperature and tactile sensation, they wore a white cotton glove. A visual metronome programmed in JavaScript was presented on a computer screen behind the participant. On each trial, this guided the researcher in delivering the strokes at one of three velocities: 0.3, 3, and 30 cm/s. Participants experienced the three velocities three times each in a pseudorandom order. On each of the nine trials, the experimenter delivered a single stroke in a distal to proximal direction over 9 cm of skin. Participants were asked to close their eyes and turn their head away while the touch was administered. After each trial, participants were presented with a fresh 20 cm long printed visual analogue scale with anchors −10 = unpleasant, 0 = neutral, and + 10 = pleasant. The participant drew a mark on the paper scale to rate the perceived pleasantness of the touch they had just experienced. After they had made their rating, the participant indicated to the experimenter they were ready to progress, and the next trial was initiated.

#### Touch Videos

Participants viewed and rated a randomized sequence of 15 short (5 s) videos (Walker et al., 2017b) depicting one individual being touched by another, at five different skin sites (back, upper arm, ventral forearm, dorsal forearm, and palm) and three different velocities (static, 3, and 30 cm/s). The clips lacked any social context, faces were not visible, and showed only the hand and forearm of one female actor “the toucher” and the relevant upper body part (back, arm, or palm) of the other male actor “the receiver.” Immediately, after viewing each clip, a new screen appeared where participants were asked to rate, on a Likert scale: how pleasant do you think that action was for the person being touched? 1 not at all – 7 extremely.

### Procedure

The participants were welcomed, seated at a desk, and given a verbal briefing, before reading the participant information sheet and signing a consent form. They then answered a series of demographic questions, presented on a computer, concerning age, gender, and childhood care status. Then, the length of their left forearm was measured to find the mid-point and two dots, 4.5 cm each side of the midpoint, were marked. On both robotic and human touch trials, touch was delivered within this 9 cm long region. Subsequently, they completed either the RTS or human touch ratings task, presentation order was counterbalanced. In the RTS condition, participants were seated in a dentist chair placing their left forearm (ventral side upward) on a memory-foam cushion attached to the arm of the chair. Participants were asked to keep their arm static and close their eyes while the RTS delivered the strokes. In the human touch condition, they sat in a desk chair with their left forearm (ventral side upward) on a memory-foam cushion attached to the chair arm. Participants were asked to keep their arm as still as possible and close their eyes while the experimenter delivered the strokes. After completing both touch tasks, participants watched and rated the touch videos. Finally, they completed the TEAQ followed by the CTQ on the computer. The videos and self-report questionnaires were presented on a computer using Qualtrics survey software (Qualtrics, Provo, UT, United States).

### Data Analysis

#### Comparison of Care Leaver and Non-care Leaver TEAQ and CTQ Scores

Touch Experiences and Attitude Questionnaire and CTQ data were analyzed using SPSS (version 24). Examination of histograms, QQ-norm plots and use of Shapiro-Wilk tests indicated the data were not normally distributed. Mann-Whitney *U* tests were therefore used to determine whether there was a significant difference between care leavers and non-care leavers for the TEAQ and CTQ subscale scores, as well as the CTQ total score. Analysis of TEAQ subscale scores required six Mann-Whitney *U* tests to be carried out; therefore, Bonferroni correction for multiple comparisons for six tests was carried out and a new alpha level for significance of 0.008 was identified. Similarly, for the CTQ analysis, there were five subscales plus the CTQ total score, so six Mann-Whitney *U* tests were carried out and a new alpha level for significance of 0.008 was identified.

#### Analysis of Ratings of Directly Experienced and Vicarious Touch

Examination of histograms and QQ-norm plots of model residuals revealed the data to be normally distributed. Furthermore, examination of fitted vs. residuals plots shows heterodescasticity was not an issue.

Since here it is assumed participants are rating pleasantness as a continuous variable and our data met the assumptions for parametric analyses (Velleman and Wilkinson, 1993; Mircioiu and Atkinson, 2017), ratings for both directly experienced touch and vicarious touch were analyzed using a linear mixed-effects model fit using the lmer function from the lme4 package (Bates et al., 2015) in R (R Core Team, 2013).

For directly experienced touch, the dependent variable was the pleasantness ratings, with fixed effects of care leaver status with two levels; care leaver and non-care leaver, velocity with three levels; 0.3, 3, and 30 cm/s, and touch modality with two levels; RTS and gloved hand. A random intercept per participant was included in the model.

For vicarious touch responses, the dependent variable was the pleasantness ratings, with fixed effects of, care leaver status with two levels; care-leaver and non-care leaver, velocity with three levels; static, 3, and 30 cm/s, and touch location with five levels; back, upper-arm, dorsal forearm, ventral forearm, and palm. A random intercept per participant was included in the model.

For both models, omnibus effects were tested using asymptotic type III Wald *χ*^2^ tests using the Anova function from the car package (Fox and Weisberg, 2019). Significant interactions were followed up using the *testInteractions* function from the phia package (De Rosario-Martinez, 2015). Simple-main effects analysis used the Holm (1979) correction for multiple comparisons.

## Results

### Analysis of CTQ Subscale Scores for Care Leavers Compared to Non-care Leavers

**Figure 1:**
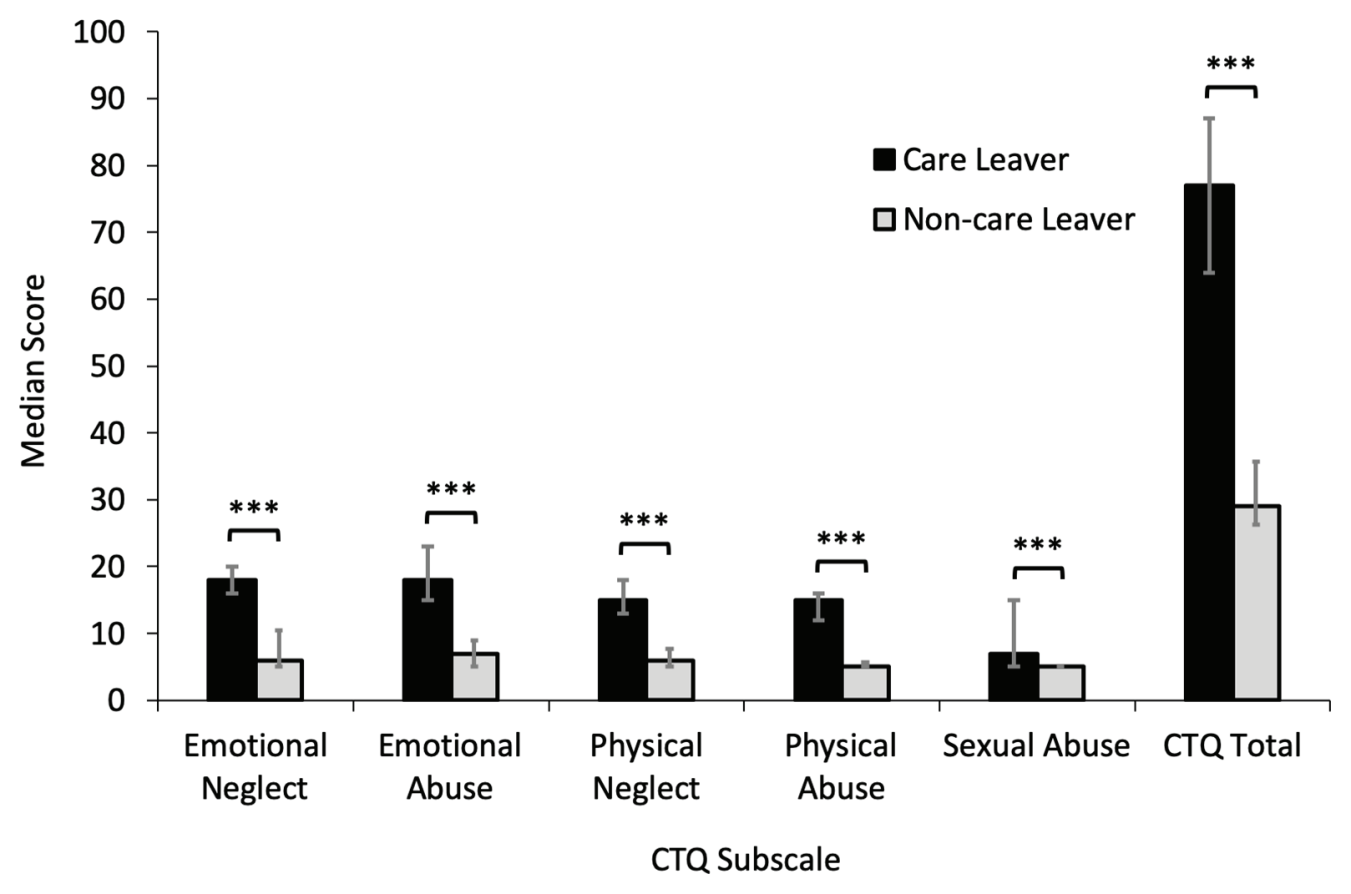
Median Childhood Touch Questionnaire (CTQ) subscale scores for care leavers and non-care leavers with error bars showing 25th and 75th percentiles. Care leavers reported significantly higher levels of all types of childhood trauma (^***^ps < 0.001), with total childhood trauma scores also significantly higher (*p* < 0.001).

Analysis of CTQ subscale scores using Mann-Whitney *U* tests to compare care leavers to non-care leavers identified that care leavers reported significantly higher levels of all types of childhood trauma, with a large effect size (emotional neglect: *U* = 40.50, *z* = −5.17, *p* < 0.001, *r* = −0.72; emotional abuse: *U* = 62.50, *z* = −4.75, *p* < 0.001, *r* = −0.67; physical neglect: *U* = 38.50, *z* = −5.25, *p* < 0.001, *r* = −0.74; physical abuse = 30.00, *z* = −5.64, *p* < 0.001, *r* = −0.79; and sexual abuse: *U* = 143.50, *z* = −3.80, *p* < 0.001, *r* = −0.53). Total CTQ scores were also significantly greater for care leavers than non-care leavers (*U* = 28.00, *z* = −5.38, *p* < 0.001, *r* = −0.75). These data are presented in Figure 1.

### Analysis of TEAQ Subscale Scores for Care Leavers Compared to Non-care Leavers

**Figure 2:**
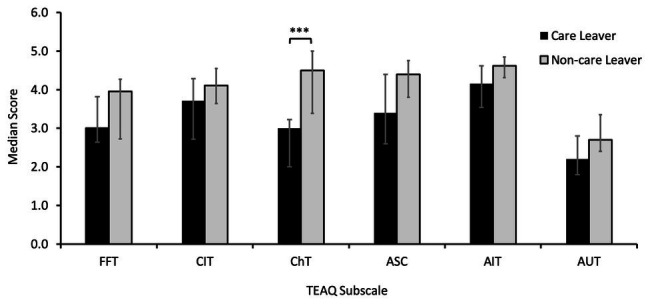
Median Touch Experiences and Attitudes Questionnaire (TEAQ) subscale scores for care leavers and non-care leavers with error bars showing 25th and 75th percentiles. Care leavers reported significantly lower levels of positive childhood touch than non-care leavers (^***^p < 0.001). No other differences were significant. FFT, friends and family touch; CIT, current intimate touch; ChT, childhood touch; ASC, attitude to self-care; AIT, attitude to intimate touch; AUT, attitude to unfamiliar touch.

Analysis of TEAQ subscale scores using Mann-Whitney *U* tests to compare care leaver to non-care leaver scores revealed care leavers reported significantly lower levels of positive childhood touch, as determined by the ChT subscale (*Mdn* = 3.00) compared to non-care leavers (*Mdn* = 4.50), with a large effect size (*U* = 106.50, *z* = −3.86, *p* < 0.001, *r* = −0.54). No significant differences between care leavers and non-care leavers for the other TEAQ subscales were identified (*ps* ≥ 0.051). These data are presented in Figure 2.

### Analysis of the Effect of Care Leaver Status on Responses to Directly Experienced Touch

There was no significant effect of touch modality (RTS vs. Gloved Hand – *χ*^2^(1) = 0.006, *p* = 0.94), nor did touch modality interact with any other factor (*ps* > 0.2).

There was a significant main effect of velocity [*χ*^2^(2) = 143.37, *p* < 0.0001] reflecting higher ratings for CT optimal 3 cm/s than 0.3 and 30 cm/s strokes (*ps* < 0.001). Ratings of 30 cm/s strokes were also significantly higher than 0.3 cm/s (*p* < 0.0001).

**Figure 3:**
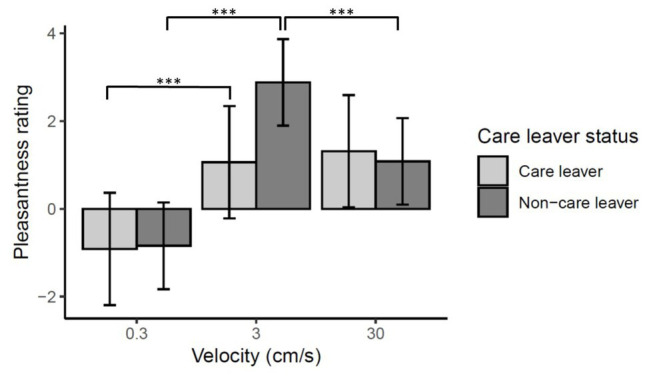
Mean pleasantness ratings ±95% confidence intervals for directly experienced touch responses. There was a significant care leaver status by velocity interaction (*p* < 0.0001) which reflects the fact care leavers appear to show less sensitivity to the specific rewarding value of CT-optimal 3 cm/s touch than the non-care leavers. Pairwise comparisons revealed that, while non-care leavers rated 3 cm/s touch significantly higher than either of the other velocities (^***^p < 0.0001), the care leavers did not rate the CT-optimal 3 cm/s touch significantly higher than 30 cm/s touch (*p* = 0.53), though they did rate it significantly more pleasant than static touch (^***^p < 0.0001). The pairwise comparison of the two groups’ ratings of 3 cm/s touch only approached significance (*p* = 0.08).

There was no main effect of care leaver status [*χ*^2^(1) = 0.52, *p* = 0.47], however, there was a significant velocity by care leaver status interaction [*χ*^2^(2) = 20.16, *p* < 0.0001]. This reflects the fact that, at a CT optimal stroking velocity of 3 cm/s, care leavers report lower levels of pleasantness than non-care leavers. While non-care leavers rated 3 cm/s touch significantly more pleasant than either of the other two velocities (*ps* < 0.0001), care leavers did not rate 3 cm/s touch any higher than 30 cm/s touch (*p* = 0.53), though they did rate it significantly higher than 0.3 cm/s touch (*p* < 0.0001). These data are summarized in Figure 3.

Analysis of simple-main effects of care leaver status for each level of velocity revealed that, after correction for multiple comparisons, the effect of care-leaver status at 3 cm/s was only marginally significant (*p* = 0.08). There was no effect of care-leaver status at either of the other velocities (*p* > 1).

### Analysis of the Effect of Care Leaver Status on Vicarious Touch Responses

**Figure 4:**
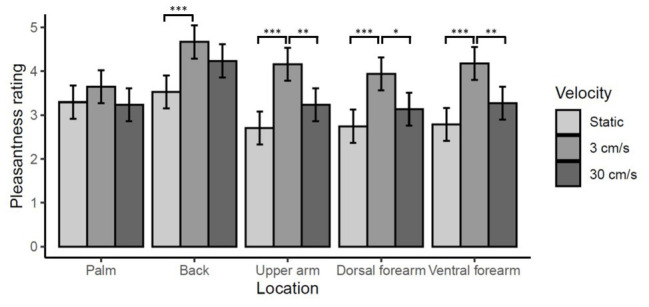
Effect of location on vicarious touch pleasantness ratings. Mean pleasantness ratings ±95% confidence intervals are shown. There is a significant location by velocity interaction (*p* < 0.01) which reflects the fact that ratings of touch on the back and on the palm show a different relationship between velocity and pleasantness than at the other three skin sites. On the palm ratings of touch pleasantness did not vary significantly by velocity (all *ps* > 0.5). On all three arm locations, 3 cm/s was rated significantly more pleasant than static or 30 cm/s touch (^***^p < 0.0001, ^**^p < 0.001, ^*^p < 0.01). While on the back ratings of 3 cm/s touch were only significantly higher than static (^***^p < 0.0001) not 30 cm/s (*p* = 0.26).

There were significant main effects of location [*χ*^2^(4) = 58.04, *p* < 0.0001], reflecting higher ratings for touch on the back than any other site (*ps* < 0.0001). There was also a significant effect of velocity [*χ*^2^(2) = 101.72, *p* < 0.0001], reflecting higher ratings of CT-optimal 3 cm/s touch (*ps* < 0.0001). 30 cm/s was also rated significantly higher than static touch (*p* < 0.0001). However, there was also a significant location by velocity interaction [*χ*^2^(8) = 17.36, *p* < 0.01]. Simple-main effects analysis of velocity for each level of location revealed a different relationship between pleasantness ratings and velocity on the back and the palm than on the other three sites. 3 cm/s was rated significantly more pleasant than either static or 30 cm/s on the upper-arm, ventral forearm, and dorsal forearm (all *ps* < 0.01), while, for the palm there were no significant differences in pleasantness across velocities (all *ps* > 0.5). On the back, 3 cm/s was rated significantly more pleasant than static (*p* < 0.0001) but not 30 cm/s (*p* = 0.26). These data are summarized in Figure 4.

**Figure 5:**
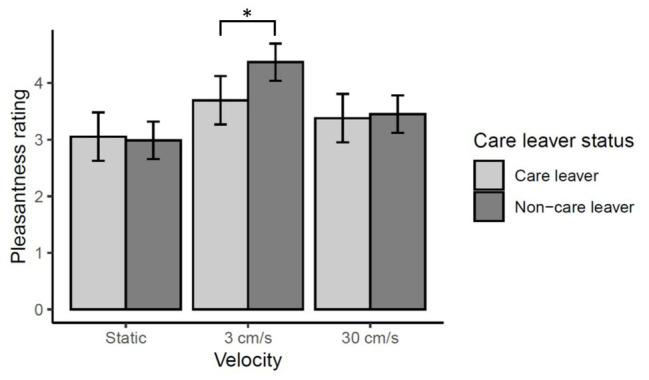
Effect of care leaver status on vicarious touch pleasantness ratings. Mean pleasantness ratings ±95% confidence intervals are shown. There was a significant care leaver status by velocity interaction (*p* = 0.0001), which reflects the fact care leavers rated touch at 3 cm/s significantly lower than non-care leavers (^*^p < 0.01). There was no significant difference between the two groups’ ratings at either of the other velocities (*ps* > 1).

There was no significant main effect of care leaver status [*χ*^2^(1) = 0.83, *p* = 0.36], nor was there a significant care leaver status by location interaction [*χ*^2^(4) = 1.23, *p* = 0.87]. However, there was a significant care leaver status by velocity interaction [*χ*^2^(2) = 15.13, *p* < 0.001]. Simple-main effects analysis of care leaver status at each level of velocity revealed that care leavers rated touch at 3 cm/s significantly lower than non-care leavers (*p* < 0.01). There were no significant differences between the two groups in their ratings of static and 30 cm/s touch (*ps* > 1). These data are summarized in Figure 5.

## Discussion

Of the young adults who took part in this study, the 19 who had spent, on average, 9 years in foster care reported significantly higher levels of abuse and neglect on all subscales of the CTQ and significantly higher levels of trauma overall than age matched controls. When asked about their experiences and attitudes toward touch, only their experiences of childhood touch were significantly more negative than the control group. This is consistent with our previous finding that scores on the Childhood Trauma subscale of the TEAQ are negatively predictive of scores on the CTQ (Trotter et al., 2018b). In line with previous reports, there were no differences in our participants’ affective ratings of robotic vs. experimenter delivered touch (Triscoli et al., 2013). This indicates both groups of participants are rating the affective quality of sensory stimulus delivered, and top-down factors such as social context are not impacting their response. However, the care leavers showed blunted sensitivity to the specific rewarding value of CT optimal 3 cm/s touch. This difference between the two groups was also apparent when participants were asked to rate vicariously experienced touch where, irrespective of touch location, the care leavers rated CT optimal 3 cm/s touch as significantly less pleasant than the control group. The observed interaction between touch pleasantness and location is consistent with our previous findings using these video stimuli and has been hypothesized to reflect variations in CT innervation density across body sites, with CTs thought to be absent from the palm and perhaps more dense in dorsal thoracic sites (Liu et al., 2007; Walker et al., 2017b). Importantly here, this pattern of ratings did not vary according to care leaver status.

Thus, consistent with our hypothesis that childhood experiences of abuse and neglect have long term consequences for the processing of socially relevant tactile stimuli, here we show that a group of young adults, reporting significantly higher levels of childhood trauma and more negative experiences of childhood touch than the control group, also showed blunted sensitivity to affective touch in our psychophysical tests. Importantly, this effect was specific. Our psychophysical measures were designed to investigate the functioning of CTs, a system of unmyelinated cutaneous C-type fibers. For both directly experienced and vicarious touch, the effects were specific to CT optimal velocity touch; we did not see a general blunting of all affective ratings.

While the neurobiological basis of the present findings is unclear, group level differences in affective touch ratings have been reported in several previous studies (Morrison et al., 2011; Crucianelli et al., 2016; Croy et al., 2016a, 2020; Sailer and Ackerley, 2017; Davidovic et al., 2018; Krahé et al., 2018) Psychologically, these reflect state (Sailer and Ackerley, 2017) and trait (Croy et al., 2016b; Crucianelli et al., 2016) differences in perception. Neurally, they have been associated with both peripheral (Morrison et al., 2011) and central (Davidovic et al., 2018) differences in the encoding of affective touch. For example, Sailer and Ackerley (2017) found that adults self-reporting a low frequency of social tactile interactions rated CT optimal touch as less pleasant than a group who reported frequent touch. However, it seems unlikely current levels of touch are driving our effects because, using our previously validated self-report measure (Trotter et al., 2018a,b), we found no difference between the two groups in their current experiences of either intimate touch or tactile interactions with friends and family.

Attachment style is a significant predictor of sensitivity to CT-optimal affective touch (Krahé et al., 2016, 2018). In their study, Krahé et al. (2018) classified participants as either securely or insecurely attached based on responses during the semi-structured, Adult Attachment Interview (AAI; George et al., 1996). Within the AAI, participants are asked, for example, to reflect on their childhood experiences including care-giver responsiveness. Those participants classified as having an insecure attachment style showed reduced discrimination of the hedonic value of CT-targeted vs. non-CT targeted, faster velocity touch. The authors propose that the enhanced sensitivity of the securely attached group to the specific rewarding value of CT targeted touch derives from a social history of seeking comfort through proximity to others. Children of mothers who provide frequent affectionate touch are more likely to be securely attached than those experiencing low levels of tactile affection (Ainsworth, 1979; Egeland and Farber, 1984; Duhn, 2010; Feldman, 2011, 2012). A secure and reinforcing relationship with a primary caregiver is a key factor predicting positive outcomes for looked-after children (Cocker and Scott, 2006). Given attachment representations are formed during early care-giving experiences and remain relatively stable across the lifespan (Waters et al., 2000), it seems possible that attachment style would mediate the observed relationship between experiences of early nurturing touch and later life sensitivity to social-affective stimuli (Walker and McGlone, 2013; McGlone et al., 2014; Fotopoulou and Tsakiris, 2017), future work should address this question.

Heart-rate variability is a biomarker of self-regulation capacity, and low levels have been linked to anxiety and depression (Friedman and Thayer, 1998; Booij et al., 2006; Melzig et al., 2009; Pappens et al., 2014; Smith et al., 2017). Maternal touch is known to have a significant influence on the developing autonomic system (Hofer, 1994; Field et al., 1995; Van Puyvelde et al., 2019b). While, both skin-to-skin contact with their mother and stroking touch increase respiratory sinus arrhythmia (RSA), a component of HRV, in young infants (Van Puyvelde et al., 2015, 2019a,b), lack of parental support is known to lead to blunted RSA development (Field et al., 1995). To the best of our knowledge, differences in somatosensory processing in those raised in foster care have not previously been reported. While the outcomes of growing up in foster care are more positive than institutionalized care, it is still a significant risk factor for poor mental-health and social isolation in later life (Stein, 2006; Gypen et al., 2017; Atkinson and Hyde, 2019), suggesting self-regulatory deficits are apparent in this group too. The blunted affective touch ratings reported in the present study provide further support for the theory that CTs are one mechanism by which early nurturing contact supports the development of an infant’s physiological and emotional regulation (Craig, 2002; Björnsdotter et al., 2010; McGlone et al., 2017; Manzotti et al., 2019; Van Puyvelde et al., 2019b).

Though the findings from the present study offer novel insight into the relationship between social history and affective touch perception, there are several limitations which should be addressed in future work. Firstly, as group, care-experienced individuals are more likely to suffer from mental health conditions than the general population (Gypen et al., 2017). In the present study, we did not take measures of past or present mental health status so cannot determine how this relates to the observed effects. However, it is noteworthy that previous studies have reported blunted ratings of affective touch were not predicted by mental health diagnosis or current affective state (Croy et al., 2016a; Sailer and Ackerley, 2017). Secondly, those who are care-experienced are less likely to go onto higher education than the general population (Evans et al., 2017). Here, our control group was primarily university students while our care leavers were not. Differences in years of education could have contributed to the differences we observed between the two groups and should be addressed in future work. Due to limitations of time and resources, our group sizes are unbalanced. While, variances between the two groups are equal and our analysis can robustly account for such differences, it may have limited our power to detect effects (Rusticus and Lovato, 2014). Finally, while the consistency in our findings between the directly felt and vicarious touch studies adds weight to the present report, in the future comparing vicarious and directly felt touch across precisely the same body locations and velocities would be of interest.

In conclusion, while studies of the impact of early life experience on later life somatosensory functioning have focused on discriminative aspects of touch (Pinkernelle et al., 2009; Nevalainen et al., 2014; Seelke et al., 2016; Nephew et al., 2017), here, we report blunted responses specifically to affective touch in young adults who have experienced early life adversity and consequently spent time in foster care. While the neurobiological basis of the observed effect remains to be explored, neural networks underpinning social and emotional functioning, within which individual differences to CT targeted touch have previously been reported, offer promising avenues for future study (Voos et al., 2013; Kaiser et al., 2015; Brauer et al., 2016; Davidovic et al., 2018; Haggarty et al., 2020). The quality of early infant-caregiver interactions predicts a child’s attachment security, emotional reactivity, and self-regulation capacity (Anderson and Keim, 2016). While national guidelines state foster parents should provide nurturing contact, local guidelines are sometimes more equivocal, and anxiety that touch could be misinterpreted means carers often admit they are wary of showing physical affection (Narey and Owers, 2018). Thus, a better understanding of the neurobiological basis of the blunted affective touch ratings we report here would reinforce the importance of providing adequate affiliative touch and help guide policies and interventions which build resilience and mitigate risk of later life adverse consequences in a vulnerable group.

## Data Availability Statement

The raw data supporting the conclusions of this article will be made available by the authors, without undue reservation.

## Ethics Statement

The studies involving human participants were reviewed and approved by Liverpool John Moores University Psychology Research Ethics Committee. The patients/participants provided their written informed consent to participate in this study.

## Author Contributions

SD and PT conceived and designed the study with input from SW, AM, and FM. AM and PT set up the laboratory, wrote the study protocols, and supervised data collection. SD and ES recruited participants and collected the data. PT and MM analyzed the data. SW, PT, and SD wrote the manuscript. All authors contributed to the article and approved the submitted version.

### Conflict of Interest

The authors declare that the research was conducted in the absence of any commercial or financial relationships that could be construed as a potential conflict of interest.
